# The Dimensions of the Aortic Valve Annulus Are Not Associated with Systolic Excursion of Its Plane in the Same Healthy Adults: Detailed Insights from the Three-Dimensional Speckle-Tracking Echocardiographic MAGYAR-Healthy Study

**DOI:** 10.3390/jcm14165760

**Published:** 2025-08-14

**Authors:** Attila Nemes, Nóra Ambrus, Csaba Lengyel

**Affiliations:** Department of Medicine, Albert Szent-Györgyi Medical School, University of Szeged, Semmelweis Street 8, P.O. Box 427, H-6725 Szeged, Hungary; ambrusnora@gmail.com (N.A.); lengyel.csaba@med.u-szeged.hu (C.L.)

**Keywords:** three-dimensional, speckle-tracking, echocardiography, aortic valve, area, excursion, annulus, healthy

## Abstract

**Background/Objectives:** The aortic valve has a prominent role in regulating blood flow and is of exceptional importance in clinical cardiological practice, as it can be affected by numerous abnormalities, so any clinical study that examines its physiological properties may be of significance. It is known that the dimensions of the aortic valve annulus (AVA) not only change during the cardiac cycle, but also undergo spatial displacement. Considering this, the question arises as to whether the AVA’s dimensions and their spatial displacement, represented by aortic annular plane systolic excursion (AAPSE), are related or not. Therefore, these parameters were simultaneously assessed using three-dimensional speckle-tracking echocardiography (3DSTE) in healthy adults. **Methods:** The present study’s cohort consisted of 148 healthy adults (mean age: 34.8 ± 12.4 years, 80 men). In all cases, two-dimensional Doppler echocardiography and 3DSTE were performed, the latter being used to assess the aortic valve. **Results:** In all subjects, end-diastolic and end-systolic AVA dimensions showed no association with an increase in AAPSE. In subjects with a greater end-diastolic AVA area (AVA-A), end-systolic AVA dimensions tended to decrease with increasing AAPSE; this trend reached statistical significance for end-systolic minimum AVA diameter, when comparing participants with AAPSE below versus above the mean. With increasing end-diastolic AVA-A, all other AVA parameters increased accordingly in all subjects and regardless of which AVA-A proved to be greater. In all subjects, and in those with a greater end-systolic AVA-A, the AAPSE proved to be similar regardless of the size of the end-diastolic AVA-A. In cases with a greater end-diastolic AVA-A, only one subject showed a very small end-diastolic AVA-A. With increasing end-systolic AVA-A, all other AVA dimensions were increased in all subjects and in cases with a greater end-diastolic or end-systolic AVA-A. AAPSE showed no significant differences between the subgroups examined, although it tended to be lower in cases with a greater end-diastolic AVA-A and the largest end-systolic AVA-A, and in subjects with a greater end-systolic AVA-A and the smallest end-systolic AVA-A. Moreover, individuals with a greater end-diastolic AVA-A and the smallest end-systolic AVA-A had a tendency for increased AAPSE. No correlations were present between AVA dimensions and AAPSE. **Conclusions:** 3DSTE-derived AVA dimensions showed no obvious associations with AAPSE in the same healthy adults.

## 1. Introduction

The aortic valve has a prominent role in regulating blood flow and is of exceptional importance in clinical cardiological practice, as it can be affected by numerous abnormalities [1,2]. Compared to those of the atrioventricular valves, the dimensions of the aortic valve annulus (AVA) are larger in about 60% of cases at the end of the systole and 32% of cases at the end of the diastole, the clinical significance of which is not yet fully understood [3,4]. In addition to the above, the AVA also undergoes spatial displacement during the cardiac cycle due to adjacent muscular areas, which can be characterized by aortic annular plane systolic excursion (AAPSE) [5,6].

In recent decades, cardiovascular imaging has undergone tremendous development, enabling detailed spatial assessment not only of the heart chambers, but also of the valves at the same time. Three-dimensional speckle-tracking echocardiography (3DSTE) is one of the most advanced imaging techniques available; it allows for rapid and accurate clinical analysis, supports both physiological and pathophysiological analyses [7,8,9,10,11], and has been found to be useful for AVA determination [3,12,13]. There are limited data on whether there is a relationship between the AVA’s dimensions and its spatial displacement (AAPSE) in healthy circumstances [5,6]. Given all these facts, a relevant question arises regarding whether the dimensions and spatial displacement of the AVA, which can be evaluated simultaneously at the same time using the same 3DSTE-derived echocardiographic datasets, are related to one another or are independent in healthy adults.

## 2. Subjects and Methods

We present the following article in accordance with the STROBE reporting checklist.

**Subject population**. The present cohort study is part of the ‘**M**otion Analysis of the heart and **G**reat vessels bY three-dimension**A**l speckle-t**R**acking echocardiography in **Healthy** subjects’ study **(MAGYAR-Healthy Study)**. This study was organized at the Department of Medicine, University of Szeged, Hungary, on the initiative of the researchers (‘Magyar’ means ‘Hungarian’ in the Hungarian language). The aims of the study were to demonstrate the clinical applicability of 3DSTE in healthy adults, validate 3DSTE measurements, determine normal reference values for measurable parameters, demonstrate new applications, and finally prove physiological associations between parameters. For these aims, more than 300 healthy individuals were recruited on voluntary bases between 2011 and 2017 and underwent physical examination, laboratory tests, standard 12-lead electrocardiography (ECG), and two-dimensional (2D) Doppler echocardiography. All the clinical evaluations performed proved to be negative, and parameters were within the normal reference ranges. None of the healthy individuals were taking any medication regularly or were obese (body mass index > 30 kg/m^2^), pregnant, athletes, yoga practitioners, or smokers. None of the subjects had a positive medical history for any known disorder or pathology.

From this pool, 148 healthy adult individuals (mean age: 34.8 ± 12.4 years, 80 men) were selected for AVA size determination and AAPSE measurement. This study was conducted in accordance with the Declaration of Helsinki (as revised in 2013), and the Institutional and Regional Human Biomedical Research Committee at the University of Szeged, Hungary, approved it under the registration number 71/2011 (latest approval 17 March 2025). Written informed consent was given by all participants.

**Two-dimensional Doppler echocardiography.** In all healthy individuals, 2D echocardiography was carried out using a Toshiba Artida^®^ echocardiographic system (Toshiba Medical Systems, Tokyo, Japan) equipped with a 1–5 MHz broadband PST-30BT phased-array transducer. Following measurement of routine left atrial and ventricular (LV) dimensions, the modified Simpsons’ method was used for the measurement of the LV ejection fraction (EF). To exclude significant valvular regurgitations or stenoses, and to assess early and late diastolic transmitral flow velocities and their ratio (E/A), Doppler echocardiography was used [14].

**Three-dimensional speckle-tracking echocardiography**. For 3DSTE studies, the same echocardiographic system was used at the same time, with the transducer switched to a 3D-capable PST-25SX matrix-array probe. After optimizing the imaging settings (gain, magnitude, etc.), 3D echocardiographic data acquisitions were carried out from the apical window. For optimal images, the subjects had to hold their breath while maintaining constant RR intervals on ECG, then 6 subvolumes over 6 cardiac cycles were acquired, and were stitched together automatically by the software. The acquired datasets were later analyzed using a dedicated vendor-provided software package, 3D Wall Motion Tracking (version 2.7, Toshiba Medical Systems, Tokyo, Japan) [7,8,9,10,11].

**Figure 1:**
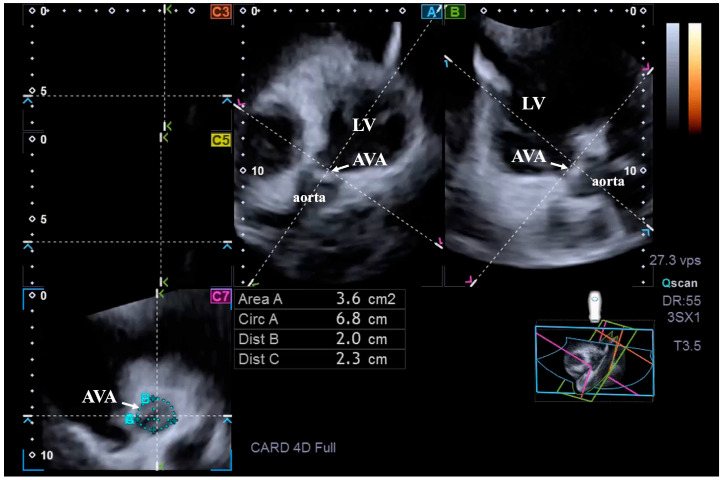
Three-dimensional speckle-tracking echocardiography-derived measurement of the aortic valve annular dimensions. Abbreviations: LV = left ventricle, AVA = aortic valve annulus, Area A = AVA area, Circ A = AVA perimeter, Dist B = minimum AVA diameter, Dist C = maximum AVA diameter.

To measure the AVA dimensions, the optimal LV longitudinal planes were first determined using apical long-axis 4-chamber and 2-chamber views. Subsequently, the aortic valve/aorta was visualized by tilting and optimizing the longitudinal planes in these long-axis views, with the planes parallel to the aortic root midline. The C7 cross-sectional view, to which the AVA was aligned, was positioned perpendicular to the longitudinal plane. Special attention had to be paid to ensure that C7 was truly perpendicular and that measurements were not taken in the Valsalva sinus or in the LV outflow tract [1,2,3]. The following AVA dimensions were assessed by direct measurement or planimetry in end-diastole and end-systole [4,12,13] (Figure 1):

- Maximum and minimum AVA diameter (AVA-Dmax and AVA-Dmin, respectively);
- AVA area (AVA-A);
- AVA perimeter (AVA-P).

Since AVA plane systolic excursion (AAPSE) represents the spatial displacement of the AVA during the cardiac cycle, following determination of the AVA in end-diastole, it was measured in end-systole, following the repeated adjustments detailed above. AAPSE was defined as the difference between the end-diastolic and end-systolic AVA locations/planes.

**Statistical analysis**. Data are presented as the mean ± standard deviation (SD) or as counts (percentages), as appropriate. The homogeneity of variances was assessed by Levene’s test. The Shapiro–Wilk test was used to test the normality of distribution. In the case of normally distributed datasets, an independent-samples *t*-test was used. In the case of not-normally distributed datasets, the Mann–Whitney-Wilcoxon test was applied. For multiple comparisons, one-way analysis of variance (ANOVA) with Bonferroni correction was used, where appropriate. For correlations, Pearson’s correlation coefficients were calculated. The Bland-Altman method was applied for intraobserver and interobserver agreements. To test the reproducibility of 3DSTE-derived AVA assessments, parameters were measured two times by the same observer (intraobserver agreement) and by two observers (interobserver agreement) in 40 healthy individuals, together with their respective interclass correlation coefficients (ICCs). Statistical significance was defined in the presence of *p* less than 0.05. All statistical analyses were performed using SPSS version 29.0.0.0. (SPSS Inc., Chicago, IL, USA).

## 3. Results

**Clinical, 2D and Doppler echocardiographic and 3DSTE data.** The clinical and 2D echocardiographic data are presented in Table 1. None of the participants had larger than/equal to grade 1 valvular regurgitation or significant stenosis in any valves. At the group level, end-systolic AVA dimensions were larger than their end-diastolic counterparts (except for AVA-Dmax) (Table 2). In 89 out of 148 subjects (60%), the end-systolic AVA area proved to be greater than the end-diastolic AVA area. In 47 participants, the end-diastolic AVA area was greater (32%), while in the remaining 8 cases (8%) end-diastolic and end-systolic AVA areas were equal.

**Classification of subjects.** Healthy adult participants were categorized based on their mean ± SD of 3DSTE-derived AAPSE, end-diastolic AVA-A, and end-systolic AVA-A. The following cut-off values, derived from the mean ± SD, were used to define subgroups:

- For AAPSE: 0.87 cm (mean − SD) and 1.45 cm (mean + SD);
- For end-diastolic AVA-A: 2.21 cm^2^ and 3.99 cm^2^, respectively;
- For end-systolic AVA-A: 2.38 cm^2^ and 4.16 cm^2^, respectively (Table 2).

Subgroup comparisons were performed for all subjects and for individuals with a greater end-systolic or end-diastolic AVA area (Table 3, Table 4 and Table 5).

**AVA dimensions in different AAPSE subgroups**. In all subjects, no association was observed between end-diastolic and end-systolic AVA dimensions with an increase in AAPSE. In subjects with a greater end-diastolic AVA-A, the end-systolic AVA dimensions showed a tendency to decrease with an increase in AAPSE; this trend reached statistical significance for end-systolic AVA-Dmin when comparing participants with AAPSE below versus above the mean. Similar finding in subjects with a greater end-systolic AVA-A could not be detected (Table 3).

**AAPSE in different end-diastolic AVA area subgroups.** With increasing end-diastolic AVA-A, all other AVA parameters showed a parallel increase in all subjects and regardless of whether the end-diastolic or end-systolic AVA-A proved to be greater. In all subjects and in cases with a greater end-systolic AVA-A, AAPSE proved to be similar regardless of the size of the end-diastolic AVA-A. In cases with a greater end-diastolic AVA-A, only one subject had a very small end-diastolic AVA-A (Table 4).

**AAPSE in different end-systolic AVA area subgroups.** With an increase in the end-systolic AVA-A, all other AVA dimensions were also increased in all subjects and in cases with a greater end-diastolic or end-systolic AVA-A. AAPSE showed no significant differences between the subgroups examined, but proved to be tendentiously reduced in cases with a greater end-diastolic AVA-A and the largest end-systolic AVA-A, and in subjects with a greater end-systolic AVA-A and the smallest end-systolic AVA-A. Moreover, individuals with greater end-diastolic AVA-A and the smallest end-systolic AVA-A had a tendency for increased AAPSE.

**Correlations.** None of the AVA dimensions showed significant correlations with AAPSE, including end-diastolic AVA-Dmax (r = 0.06, *p* = 0.47), AVA-Dmin (r = 0.03, *p* = 0.77), AVA-A (r = 0.06, *p* = 0.45), and AVA-P (r = 0.08, *p* = 0.36) as well as end-systolic AVA-Dmax (r = −0.01, *p* = 0.88), AVA-Dmin (−0.07, *p* = 0.38), AVA-A (r = 0.04, *p* = 0.65), and AVA-P (r = 0.04, *p* = 0.61).

**Reproducibility of 3DSTE-derived AVA assessments.** Intraobserver and interobserver agreements of end-diastolic and end-systolic AVA diameters, areas, and perimeters are presented with their respective ICCs in Table 6.

## 4. Discussion

Today, the most advanced cardiovascular imaging techniques allow for the simultaneous spatial evaluation of multiple cardiac chambers and valves with high accuracy and feasibility. One of the best examples of this is 3DSTE, which combines the advantages of 3D echocardiography and STE, and which, in addition to being suitable for the volumetric and functional assessment of cardiac chambers, appears to be suitable for quantifying valvular annuli and their spatial displacement. The aortic valve has been less studied with 3DSTE, although normal references of its annular dimensions are defined in the literature [4,12,13].

The aortic valve is a tri-leaflet valve with a fibrous annulus located between the aorta and the LV, and it ensures unidirectional blood flow under physiologic conditions. Due to adjacent muscular structures, it exhibits both dimensional changes and spatial displacement, represented by AAPSE, during the cardiac cycle. However, according to the literature, only two-thirds of cases have a greater end-systolic than end-diastolic AVA-A; in most of the remaining subjects, the AVA-A is larger in end-diastole than in end-systole [3,4]. The clinical significance of the aortic valve and AVA lies in the fact that in the presence of various pathologies, its involvement can have a significant impact on blood flow and, secondarily, on myocardial function [1,2,15,16], which can be assessed by non-invasive imaging methods [16,17,18,19,20], like 3DSTE [4,12,13]. The present study aimed to assess whether AVA dimensions and systolic excursion of its plane, represented by AAPSE, show any associations in healthy adults at the group level, and whether the end-systolic or end-diastolic AVA-A is larger.

The present study has several implications. Firstly, it has been demonstrated that AVA dimensions and its spatial displacement (AAPSE) can be assessed by 3DSTE during the cardiac cycle at the same time, using the same acquired 3D echocardiographic dataset. This sort of approach enables their simultaneous analysis, allowing for (patho)physiologic studies. Secondly, AVA dimensions and AAPSE showed no associations or correlations, even after subgroup analyses. This remained true for cases with either a greater end-systolic AVA-A (majority of cases) or a greater end-diastolic AVA-A (minority of cases). However, a tendency for reduced AAPSE could be demonstrated in two subgroups—a very small number of cases with a greater end-diastolic AVA-A and the largest end-systolic AVA-A (2% of cases), and cases with a greater end-systolic AVA-A and the smallest end-systolic AVA-A (5% of cases). Moreover, in individuals with a greater end-diastolic AVA-A and the smallest end-systolic AVA-A (7% of cases), a tendency for increased AAPSE was found. All of these findings suggest the need for further analysis in these special groups of subjects to confirm the presented results. Thirdly, these findings raise clinically relevant questions. The first important question is whether, in the presence of certain pathologies, the proportion of patients with a higher end-diastolic AVA-A is greated than the proportion of those with higher end-systolic AVA-A. In healthy adults, the proportion of individuals with this characteristic was found to be appr. 30% [3,4]. It is not clear whether this has any clinical or prognostic impact for patients. There is also the question of what abnormalities (decreases) the AAPSE shows in the presence of certain pathologies, in addition to the above, and whether this has clinical significance. For instance, what abnormalities are present in aortic valve stenosis, and is an improvement in the values of the above parameters expected following an invasive procedure? Furthermore, the present study did not show a relationship between AVA dimensions and AAPSE. It may also be questioned whether the above-mentioned theoretical abnormalities affect the relationship between AVA dimensions and AAPSE.

**Limitations section.** Several limitations should be acknowledged in this study:

- The image quality observed using 3DSTE was inferior to that of 2D echocardiography, which may be due to its lower spatial and temporal resolution, transducer size, etc., limiting its clinical applicability [7,8,9,10,11].
- In the present study, the measurement accuracy of 3DSTE was not compared with that of gold-standard technologies such as cardiac computer tomography or magnetic resonance imaging; accordingly, the absolute reliability of the AVA size and AAPSE data was not confirmed, which may indicate technical and methodological errors. However, the usefulness of 3D echocardiography in the assessment of AVA dimensions has already been demonstrated in the literature [16].
- Although 3DSTE is suitable for mitral and tricuspid annular assessments in addition to volumetric and functional measurements of the heart chambers, such analyses were beyond the scope of this study [21].
  Although 3DSTE is suitable for mitral and tricuspid annular assessments in addition to volumetric and functional measurements of the heart chambers, such analyses were beyond the scope of this study [21].
- We did not aim to assess AVA dimensions and their spatial displacement by any other imaging techniques as well, such as STE or cardiac magnetic resonance imaging. However, this could be a topic of further investigations.
- Moreover, the study did not investigate the complex associations between the AVA and the ascending aorta.
- Gender-based comparisons were not performed due to the relatively limited sample size.
- From a statistical point of view, there was potential for selection bias, misclassification bias, and residual, unmeasured, time-varying confounding, type II errors (power), which could limit the usability and reliability of the findings.

## 5. Conclusions

3DSTE-derived AVA dimensions showed no obvious associations with AAPSE in the same healthy adults. The results may raise the question of whether the determination of the end-diastolic and end-systolic size of the AVA and its spatial displacement, as characterized by AAPSE, have clinical and prognostic significance in certain pathological conditions.

## Figures and Tables

**Table 1:** Clinical and two-dimensional echocardiographic data.

Data	Measures
**Clinical data**	
** *n***	148
** Mean age (years)**	34.8 ± 12.4
** Males (%)**	80 (54)
** Systolic blood pressure (mmHg)**	119 ± 3
** Diastolic blood pressure (mmHg)**	79 ± 4
** Heart rate (1/s)**	71 ± 3
** Height (cm)**	72.7 ± 16.9
** Weight (kg)**	172.1 ± 11.6
**Two-dimensional echocardiographic data**	
** LA diameter (mm)**	37.5 ± 3.7
** LV end-diastolic diameter (mm)**	48.3 ± 3.6
** LV end-systolic diameter (mm)**	32.1 ± 3.1
** LV end-diastolic volume (mL)**	106.5 ± 24.0
** LV end-systolic volume (mL)**	37.8 ± 9.0
** Interventricular septum (mm)**	9.2 ± 1.3
** LV posterior wall (mm)**	9.4 ± 1.5
** LV ejection fraction (%)**	64.9 ± 3.9
** Early diastolic mitral inflow velocity—E (cm/s)**	78.6 ± 15.7
** Late diastolic mitral inflow velocity—A (cm/s)**	59.3 ± 14.2

**Abbreviations:** LA = left atrial, LV = left ventricular.

**Table 2:** Three-dimensional speckle-tracking echocardiography-derived aortic valve annular dimensions.

Parameters	Measures
**End-diastolic maximum aortic valve annular diameter (AVA-Dmax-D, cm)**	1.98 ± 0.34
**End-diastolic minimum aortic valve annular diameter (AVAD-Dmin-D, cm)**	1.79 ± 0.29 *
**End-diastolic aortic valve annular area (AVA-A-D, cm^2^)**	3.10 ± 0.89 *
**End-diastolic aortic valve annular perimeter (AVA-P-D, cm)**	6.21 ± 0.94 *
**End-systolic aortic valve annular diameter (AVA-Dmax-S, cm)**	2.02 ± 0.32
**End-systolic aortic valve annular diameter (AVA-Dmin-S, cm)**	1.84 ± 0.29
**End-systolic aortic valve annular area (AVA-A-S, cm^2^)**	3.27 ± 0.89
**End-systolic aortic valve annular perimeter (AVA-P-S, cm)**	6.42 ± 0.89
**Aortic valve annular plane systolic excursion (AAPSE, cm)**	1.16 ± 0.29

* *p* < 0.05 vs. end-systolic counterpart.

**Table 3:** Aortic valve annular dimensions and systolic excursion of its plane in different aortic valve annular plane systolic excursion groups.

	AAPSE ≤ 0.87 cm (*n* = 21)	0.87 cm < AAPSE < 1.45 cm (*n* = 104)	1.45 cm ≤ AAPSE (*n* = 23)	AAPSE ≤ 0.87 cm (*n* = 6)	0.87 cm < AAPSE < 1.45 cm (*n* = 32)	1.45 cm ≤ AAPSE (*n* = 9)	AAPSE ≤ 0.87 cm (*n* = 12)	0.87 cm < AAPSE < 1.45 cm (*n* = 64)	1.45 cm ≤ AAPSE (*n* = 13)
	All (*n* = 148)			Greater End-Diastolic AVA Area (*n* = 47)			Greater End-Systolic AVA Area (*n* = 89)		
**AVA-Dmax-D (cm)**	1.96 ± 0.27	1.99 ± 0.34	1.97 ± 0.39	2.13 ± 0.29	2.10 ± 0.30 *	2.04 ± 0.31 *	1.88 ± 0.23 *	1.94 ± 0.35 *	1.91 ± 0.44 *
**AVA-Dmin-D (cm)**	1.80 ± 0.23 *	1.78 ± 0.31 *	1.82 ± 0.30	1.93 ± 0.12	1.90 ± 0.25 *	1.87 ± 0.21 *	1.77 ± 0.26	1.73 ± 0.32 *	1.78 ± 0.35
**AVA-A-D (cm^2^)**	3.00 ± 0.74 *	3.10 ± 0.93 *	3.18 ± 0.84	3.57 ± 0.41 *	3.46 ± 0.91 *	3.41 ± 0.73 *	2.81 ± 0.77 *	2.94 ± 0.92 *	2.98 ± 0.88 *
**AVA-P-D (cm)**	6.17 ± 0.78 *	6.19 ± 0.99 *	6.35 ± 0.83	6.78 ± 0.44 *	6.43 ± 1.08	6.63 ± 0.66 *	5.95 ± 0.80 *	6.09 ± 0.95 *	6.12 ± 0.88 *
**AVA-Dmax-S (cm)**	2.02 ± 0.22	2.03 ± 0.32	2.02 ± 0.35	2.07 ± 0.16	1.93 ± 0.27	1.86 ± 0.31	2.02 ± 0.26	2.08 ± 0.34	2.12 ± 0.35
**AVA-Dmin-S (cm)**	1.86 ± 0.24	1.84 ± 0.30	1.80 ± 0.29	2.00 ± 0.12	1.79 ± 0.30	1.70 ± 0.26 †	1.84 ± 0.26	1.87 ± 0.30	1.85 ± 0.30
**AVA-A-S (cm^2^)**	3.16 ± 0.69	3.27 ± 0.93	3.34 ± 0.84	3.35 ± 0.49	2.96 ± 0.80	2.92 ± 0.69	3.19 ± 0.76	3.48 ± 0.96	3.60 ± 0.85
**AVA-P-S (cm)**	6.35 ± 0.70	6.42 ± 0.92	6.50 ± 0.85	6.55 ± 0.52	6.14 ± 0.85	6.10 ± 0.86	6.36 ± 0.77	6.61 ± 0.93	6.75 ± 0.77
**AAPSE (cm)**	0.70 ± 0.11	1.15 ± 0.16 †	1.61 ± 0.15 †‡	0.78 ± 0.04	1.18 ± 0.15 †	1.61 ± 0.15 †‡	0.64 ± 0.11	1.14 ± 0.16 †	1.62 ± 0.15 †‡

Independent-samples *t*-tests, the Mann–Whitney–Wilcoxon test, and ANOVA were used as statistical methods. Abbreviations. AVA = aortic valve annulus; Dmax = maximum diameter; Dmin = minimum diameter; A = area; P = perimeter; S = end-systolic; D = end-diastolic; AAPSE = aortic valve annular plane systolic excursion; * *p* < 0.05 vs. AVA counterpart; † *p* < 0.05 vs. mean − SD AAPSE; ‡ *p* < 0.05 vs. mean AAPSE.

**Table 4:** Aortic valve annular dimensions and systolic excursion of its plane in different end-diastolic aortic valve annular area groups.

	AVA-A-D ≤ 2.21 cm^2^ (*n* = 22)	2.21 cm^2^ < AVA-A-D < 3.99 cm^2^ (*n* = 103)	3.99 cm^2^ ≤ AVA-A-D (*n* = 23)	AVA-A-D ≤ 2.21 cm^2^ (*n* = 1)	2.21 cm^2^ < AVA-A-D < 3.99 cm^2^ (*n* = 35)	3.99 cm^2^ ≤ AVA-A-D (*n* = 11)	AVA-A-D ≤ 2.21 cm^2^ (*n* = 19)	2.21 cm^2^ < AVA-A-D < 3.99 cm^2^ (*n* = 58)	3.99 cm^2^ ≤ AVA-A-D (*n* = 12)
	All (*n* = 148)			Greater End-Diastolic AVA Area (*n* = 47)			Greater End-Systolic AVA Area (*n* = 89)		
**AVA-Dmax-D (cm)**	1.52 ± 0.22 *	1.98 ± 0.23 †	2.43 ± 0.23 †‡	1.4	2.02 ± 0.25 *	2.38 ± 0.20 *‡	1.53 ± 0.22 *	1.94 ± 0.21 *†	2.48 ± 0.26 †‡
**AVA-Dmin-D (cm)**	1.34 ± 0.12 *	1.80 ± 0.19 †	2.20 ± 0.15 †‡	1.4	1.83 ± 0.18 *	2.16 ± 0.14 ‡	1.33 ± 0.11 *	1.78 ± 0.19 *†	2.23 ± 0.15 †‡
**AVA-A-D (cm^2^)**	1.78 ± 0.32 *	3.05 ± 0.44 *†	4.57 ± 0.59 †‡	1.6	3.16 ± 0.45 *	4.61 ± 0.61 *‡	1.76 ± 0.32 *	2.98 ± 0.39 *†	4.53 ± 0.58 *†‡
**AVA-P-D (cm)**	4.83 ± 0.51 *	6.24 ± 0.45 *†	7.38 ± 1.16 †‡	4.6	6.37 ± 0.48 *	7.12 ± 1.56 ‡	4.82 ± 0.54 *	6.17 ± 0.39 *†	7.63 ± 0.48 *†‡
**AVA-Dmax-S (cm)**	1.70 ± 0.24	2.02 ± 0.27 †	2.35 ± 0.25 †‡	1.3	1.88 ± 0.26	2.16 ± 0.15 ‡	1.73 ± 0.22	2.11 ± 0.25 †	2.52 ± 0.20 †‡
**AVA-Dmin-S (cm)**	1.54 ± 0.16	1.83 ± 0.25 †	2.13 ± 0.25 †‡	1.3	1.74 ± 0.25	2.02 ± 0.27 ‡	1.57 ± 0.15	1.88 ± 0.25 †	2.24 ± 0.18 †‡
**AVA-A-S (cm^2^)**	2.30 ± 0.41	3.23 ± 0.69 †	4.36 ± 0.82 †‡	1.5	2.80 ± 0.59	3.78 ± 0.64 ‡	2.37 ± 0.39	3.52 ± 0.62 †	4.89 ± 0.56 †‡
**AVA-P-S (cm)**	5.39 ± 0.51	6.41 ± 0.69 †	7.50 ± 0.69 †‡	4.3	5.98 ± 0,67	7.02 ± 0.60 ‡	5.47 ± 0.47	6.69 ± 0.56 †	7.93 ± 0.45 †‡
**AAPSE (cm)**	1.10 ± 0.25	1.17 ± 0.31	1.14 ± 0.24	0.9	1.24 ± 0.29	1.14 ± 0.19	1.13 ± 0.25	1.14 ± 0.32	1.15 ± 0.28

Independent-samples *t*-tests, the Mann–Whitney–Wilcoxon test, and ANOVA were used as statistical methods. Abbreviations. AVA = aortic valve annulus; Dmax = maximum diameter; Dmin = minimum diameter; A = area; P = perimeter; S = end-systolic; D = end-diastolic; AAPSE = aortic valve annular plane systolic excursion; * *p* < 0.05 vs. AVA counterpart; † *p* < 0.05 vs. mean − SD AAPSE; ‡ *p* < 0.05 vs. mean AAPSE.

**Table 5:** Aortic valve annular dimensions and systolic excursion of its plane in different end-systolic aortic valve annular area groups.

	AVA-A-S ≤ 2.38 cm^2^ (*n* = 21)	2.38 cm^2^ < AVA-A-S < 4.16 cm^2^ (*n* = 102)	4.16 cm^2^ ≤ AVA-A-S (*n* = 25)	AVA-A-S ≤ 2.38 cm^2^ (*n* = 10)	2.38 cm^2^ < AVA-A-S < 4.16 cm^2^ (*n* = 34)	4.16 cm^2^ ≤ AVA-A-S (*n* = 3)	AVA-A-S ≤ 2.38 cm^2^ (*n* = 8)	2.38 cm^2^ < AVA-A-S < 4.16 cm^2^ (*n* = 59)	4.16 cm^2^ ≤ AVA-A-S (*n* = 22)
	All (*n* = 148)			Greater End-Diastolic AVA Area (*n* = 47)			Greater End-Systolic AVA Area (*n* = 89)		
**AVA-Dmax-D (cm)**	1.63 ± 0.25	1.97 ± 0.28 †	2.32 ± 0.29 *†‡	1.75 ± 0.17 *	2.16 ± 0.24 *†	2.53 ± 0.05 †‡	1.51 ± 0.29	1.85 ± 0.25 *†	2.30 ± 0.29 *†‡
**AVA-Dmin-D (cm)**	1.46 ± 0.18	1.78 ± 1.24 *†	2.12 ± 0.22 *†‡	1.59 ± 0.10 *	1.94 ± 0.16 *†	2.30 ± 0.14 †‡	1.30 ± 0.10 *	1.67 ± 0.22 *†	2.10 ± 0.22 *†‡
**AVA-A-D (cm^2^)**	2.06 ± 0.48	3.07 ± 0.71 *†	4.09 ± 0.76 *†‡	2.42 ± 0.30 *	3.66 ± 0.64 *†	4.77 ± 0.37 †‡	1.59 ± 0.33 *	2.71 ± 0.52 *†	4.00 ± 0.75 *†‡
**AVA-P-D (cm)**	5.14 ± 0.65	6.18 ± 0.78 *†	7.22 ± 0.68 *†‡	5.57 ± 0.35 *	6.67 ± 0.89 †	7.83 ± 0.25 †‡	4.59 ± 0.63	5.88 ± 0.57 *†	7.13 ± 0.67 *†‡
**AVA-Dmax-S (cm)**	1.59 ± 0.20	2.01 ± 0.21 †	2.45 ± 0.21 †‡	1.56 ± 0.21	2.01 ± 0.18 †	2.30 ± 0.14 †‡	1.58 ± 0.11	2.00 ± 0.23 †	2.47 ± 0.20 †‡
**AVA-Dmin-S (cm)**	1.45 ± 0.16	1.82 ± 0.20 †	2.22 ± 0.18 †‡	1.40 ± 0.19	1.86 ± 0.18 †	2.27 ± 0.17 †‡	1.50 ± 0.10	1.78 ± 0.21 †	2.22 ± 0.18 †‡
**AVA-A-S (cm^2^)**	1.96 ± 0.28	3.19 ± 0.45 †	4.70 ± 0.50 †‡	1.91 ± 0.31	3.19 ± 0.40 †	4.53 ± 0.40 †‡	1.96 ± 0.22	3.19 ± 0.45 †	4.73 ± 0.51 †‡
**AVA-P-S (cm)**	5.01 ± 0.39	6.39 ± 0.47 †	7.75 ± 0.43 †‡	4.95 ± 0.44	6.42 ± 0.44 †	7.67 ± 0.25 †‡	5.01 ± 0.33	6.38 ± 0.47 †	7.76 ± 0.45 †‡
**AAPSE (cm)**	1.16 ± 0.25	1.16 ± 0.31	1.15 ± 0.23	1.32 ± 0.20	1.19 ± 0.29	1.03 ± 0.21	1.05 ± 0.22	1.15 ± 0.33	1.16 ± 0.23

Independent-samples *t*-tests, the Mann–Whitney–Wilcoxon test, and ANOVA were used as statistical methods. Abbreviations. AVA = aortic valve annulus; Dmax = maximum diameter; Dmin = minimum diameter; A = area; P = perimeter; S = end-systolic; D = end-diastolic; AAPSE = aortic valve annular plane systolic excursion; * *p* < 0.05 vs. AVA counterpart; † *p* < 0.05 vs. mean − SD AAPSE; ‡ *p* < 0.05 vs. mean AAPSE.

**Table 6:** Intra- and interobserver variability for three-dimensional speckle-tracking echocardiography-derived aortic valve annular dimensions and systolic excursion of its plane.

	Intraobserver Agreement		Interobserver Agreement	
	Mean ± 2SD Difference in Values Obtained by 2 Measurements by the Same Observer	ICC Between Measurements by the Same Observer	Mean ± 2SD Difference in Values Obtained by 2 Observers	ICC Between Independent Measurements of 2 Observers
**AVA-Dmax-D (cm)**	−0.04 ± 0.21	0.88 (*p* < 0.01)	−0.05 ± 0.16	0.88 (*p* < 0.01)
**AVA-Dmin-D (cm)**	−0.02 ± 0.22	0.91 (*p* < 0.01)	−0.04 ± 0.22	0.93 (*p* < 0.01)
**AVA-A-D (cm^2^)**	−0.11 ± 0.61	0.93 (*p* < 0.01)	−0.10 ± 0.56	0.94 (*p* < 0.01)
**AVA-P-D (cm)**	−0.06 ± 0.62	0.90 (*p* < 0.01)	−0.11 ± 0.68	0.94 (*p* < 0.01)
**AVA-Dmax-S (cm)**	0.02 ± 0.32	0.92 (*p* < 0.01)	0.03 ± 0.30	0.94 (*p* < 0.01)
**AVA-Dmin-S (cm)**	0.06 ± 0.33	0.82 (*p* < 0.01)	0.03 ± 0.36	0.83 (*p* < 0.01)
**AVA-A-S (cm^2^)**	0.11 ± 0.68	0.91 (*p* < 0.01)	0.12 ± 0.76	0.94 (*p* < 0.01)
**AVA-P-S (cm)**	−0.02 ± 0.56	0.90 (*p* < 0.01)	0.03 ± 0.52	0.92 (*p* < 0.01)
**AAPSE (cm)**	−0.02 ± 0.18	0.91 (*p* < 0.01)	−0.03 ± 0.18	0.91 (*p* < 0.01)

Abbreviations. ICC = interclass correlation coefficient; AVA = aortic valve annulus; Dmax = maximum diameter; Dmin = minimum diameter; A = area; P = perimeter; S = end-systolic; D = end-diastolic; AAPSE = aortic valve annular plane systolic excursion.

## Data Availability

The data presented in this study are available on request from the corresponding author.
